# Calcareous sponge genomes reveal complex evolution of α-carbonic anhydrases and two key biomineralization enzymes

**DOI:** 10.1186/s12862-014-0230-z

**Published:** 2014-11-25

**Authors:** Oliver Voigt, Marcin Adamski, Kasia Sluzek, Maja Adamska

**Affiliations:** Department of Earth and Environmental Sciences, Ludwig-Maximilians-Universität München, Richard-Wagner-Street 10, 80333 München, Germany; Sars International Centre for Marine Molecular Biology, University of Bergen, Thormøhlensgt. 55, Bergen, 5008 Norway

**Keywords:** Alpha carbonic anhydrase, Calcareous sponges, Biomineralization, Evolution

## Abstract

**Background:**

Calcium carbonate biominerals form often complex and beautiful skeletal elements, including coral exoskeletons and mollusc shells. Although the ability to generate these carbonate structures was apparently gained independently during animal evolution, it sometimes involves the same gene families. One of the best-studied of these gene families comprises the α- carbonic anhydrases (CAs), which catalyse the reversible transformation of CO_2_ to HCO_3_^−^ and fulfill many physiological functions. Among Porifera –the oldest animal phylum with the ability to produce skeletal elements– only the class of calcareous sponges can build calcitic spicules, which are the extracellular products of specialized cells, the sclerocytes. Little is known about the molecular mechanisms of their synthesis, but inhibition studies suggest an essential role of CAs. In order to gain insight into the evolution and function of CAs in biomineralization of a basal metazoan species, we determined the diversity and expression of CAs in the calcareous sponges *Sycon ciliatum* and *Leucosolenia complicata* by means of genomic screening, RNA-Seq and RNA *in situ* hybridization expression analysis. Active biomineralization was located with calcein-staining.

**Results:**

We found that the CA repertoires of two calcareous sponge species are strikingly more complex than those of other sponges. By characterizing their expression patterns, we could link two CAs (one intracellular and one extracellular) to the process of calcite spicule formation in both studied species. The extracellular biomineralizing CAs seem to be of paralogous origin, a finding that advises caution against assuming functional conservation of biomineralizing genes based upon orthology assessment alone. Additionally, calcareous sponges possess acatalytic CAs related to human CAs X and XI, suggesting an ancient origin of these proteins. Phylogenetic analyses including CAs from genomes of all non-bilaterian phyla suggest multiple gene losses and duplications and presence of several CAs in the last common ancestor of metazoans.

**Conclusions:**

We identified two key biomineralization enzymes from the CA-family in calcareous sponges and propose their possible interaction in spicule formation. The complex evolutionary history of the CA family is driven by frequent gene diversification and losses. These evolutionary patterns likely facilitated the numerous events of independent recruitment of CAs into biomineralization within Metazoa.

**Electronic supplementary material:**

The online version of this article (doi:10.1186/s12862-014-0230-z) contains supplementary material, which is available to authorized users.

## Background

Carbonate skeletons are formed in many animal phyla. The ability to form calcium carbonate skeletal elements apparently evolved several times independently, but, nonetheless, a core set of certain genes seems to be involved in carbonate biomineralization in different animal groups [1,2]. Components of this ‘biomineralization toolkit’ could already have been present in the last common ancestor of Metazoa, or gained their biomineralizing function several times independently from suitable precursor proteins [2]. One of the best-studied components of the ‘biomineralization toolkit’ [3-6] is probably the gene family of α-carbonic anhydrases (CAs). CAs are metalloenzymes requiring zinc, which is usually bound by three distinct histidine residues; the proteins catalyse the reversible reaction of CO_2_ and water to HCO_3_^−^ and H^+^ [7]. With this function CAs also are involved in a number of other metabolic processes, such as CO_2_- transport or pH- and ion-regulation [8,9], and different CAs with specific functions are usually present in a species’ genome. In mammals, for example, there are up to 16 CAs (including three acatalytic forms referred to as CA-related proteins or CARPs), which are secreted, membrane-bound, cytosolic or mitochondrial proteins [10-12]. Specialized forms of CAs have been shown to be key elements in the formation of carbonate skeletons in many different invertebrate animal phyla, including sponges [3,13-16]. Jackson and co-workers [3] reported the involvement of CA in the formation of the basal carbonate skeleton of the demosponge *Astrosclera willeyana*. While several other sponges can form such basal carbonate skeletons in addition to or in place of their siliceous spicules, only sponges from one of the four currently recognized sponge classes, the calcareous sponges (Class Calcarea), are capable of producing calcite spicules, which is a synapomorphy of this class [17]. The calcite spicules of Calcarea constitute a substantial part of their body weight and, by supporting the soft tissue, enable the growth of larger sponge bodies. Therefore, calcite spicule formation has to be considered a key innovation of this sponge group, which triggered the radiation of calcarean diversity we observe today.

**Figure 1:**
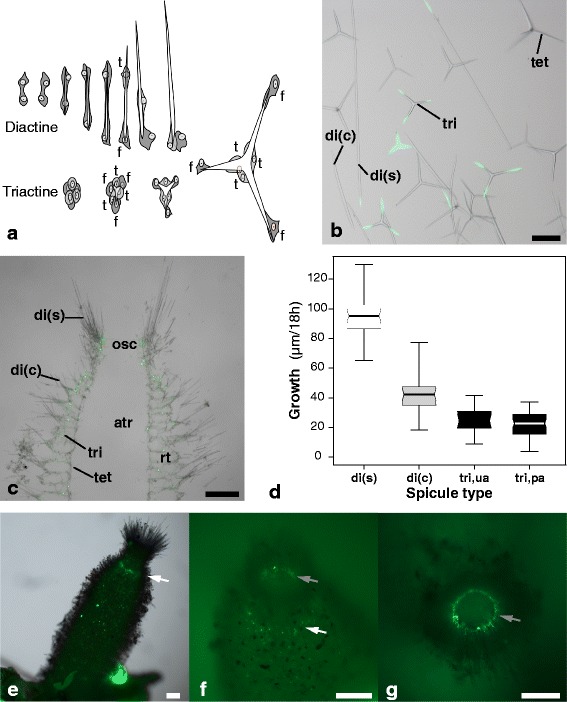
**Spicules and their formation in** ***S. ciliatum*** **. (a)** Formation of diactines and triactines by sclerocytes in calcareous sponges (redrawn from [18,19]). **(b)** isolated spicules; scale bar: 100 μm. **(c)** skeletal arrangement; scale bar: 250 μm. **(d)** Spicule growth in 18 h observed in three spicule types of *S. ciliatum*. **(e-g)** Location of spicule formation in *S. ciliatum* (calcein disodium staining). Single spicules are formed all over the sponge body, with two regions of denser spicule formation: (1) the radial tube formation zone (white arrow, e) and the proximal tips of the slender diactines of the osculum (inside the osculum, grey arrow: f, g); scale bars: 250 μm. b,c,e: light microscopic images overlayed with fluorescence microscope images. f,g: fluorescence microscope images. Abbreviations: di(c) curved diactines from the distal end of the radial tubes; di(s): slender diactines of the oscular fringe; f: founder cell; t: thickener cell; tri: triactines; tet: tetractines.

Depending on the number of rays, the calcitic spicules can be categorized into diactines, triactines and tetractines [17], which are formed respectively by two, six or seven specialized cells, the sclerocytes [18-20]. Each spicule grows in an organic sheath of unknown composition, within an extracellular space initially sealed by septate junctions between the involved sclerocytes [21]. The secretory activity of these cells and their movement controls spicule growth (Figure 1a [20]). Among sclerocytes, the so-called “founder cell” promotes growth of the actine tip, while deposits from the “thickener cell” thicken the spicule [18-20]. Little is known about the molecular mechanisms of spicule formation by sclerocytes. However, as in other invertebrates, CA seems to play an important role in this process in calcareous sponges: Spicule formation is ceased or reduced by the application of specific CA-inhibitors to living calcareous sponges [22], but the CAs were not characterized. Attempts to extract or characterize CA-proteins from the calcareous sponge *S. ciliatum* have not been successful [22]. Only recently, a CA of another calcareous sponge has been described and a role in spicule formation and dissolution proposed [23,24]. However, various CAs can usually be found in metazoan genomes, and often more than one CA can be linked to biomineralization in corals (e.g. [11,25]), molluscs (e.g. [6,26]) and urchins (e.g. [4,13]).

In this study, we aimed to describe the CA-repertoire of calcareous sponges and identify the CAs involved in spicule formation in order to gain further insight into the evolution of carbonate biomineralization in non-bilaterian animals. We investigated the CAs present in the genome and transcriptome of the emerging model system *Sycon ciliatum* (Class Calcarea, Subclass Calcaronea, Order Leucosolenida, Family Sycettidae) and a second species, *Leucosolenia complicata* (Class Calcarea, Subclass Calcaronea, Order Leucosolenida, Family Leucosoleniidae) [27-29]*.* Active biomineralization was detected by calcein staining methods and correlated with expression data from RNA in-situ hybridization and RNA-seq analyses. Phylogenetic analyses with CAs from genomes of all non-bilaterian phyla let us draw conclusions about the evolution of CA proteins in calcareous sponges and in Metazoa in general.

## Results

### Calcein staining experiments

*S. ciliatum* is a typical syconoid sponge, with a tube-like body and an apical oscular opening. In the oscular region, the sponge wall is thin. Below this, the sponge wall widens, with tubes arranged radially around the central atrial cavity, the so-called radial tubes. Four spicule types can readily be distinguished in the species (e.g. [18]): (1) long, slender diactines, forming a palisade-like fringe around the osculum; (2) smaller, curved diactines, occurring as tufts on the distal ends of the radial tubes; (3) triactines, supporting the radial tubes and the atrial wall; (4) tetractines, supporting the atrial wall, with a forth ray reaching into the central cavity (Figure 1b, c). Growing spicules were detected by exposing live *S. ciliatum* sponges to a calcein disodium solution in seawater for 3, 18 or 24 h. We observed isolated spicules, sections and complete specimens from these treatments (Figure 1b-c). Isolated spicules from calcein treated sponges largely confirmed results from another *Sycon* species [30] and observations of spicule formation by Woodland [18] and Minchin [19]. Results show that after an initial phase diactine growth is restricted to the proximal actine, and triactines and tetractines grow at their tips, in both cases due to the activity of the founder cells (Figure 1a,b). A second band of calcite deposition on diactines, previously reported and interpreted as the thickening activity of later stage diactines [30], could not be observed. Labeled triactines of *S. ciliatum* sometimes provided a previously undocumented calcite precipitation pattern*.* In triactines, distinctions between the so-called unpaired actine, pointing to the distal end of the radial tube, and the two paired actines can be made (Additional file 1). The angle between the paired actines differs from the angle between the paired and the unpaired actines. Also, frequently, a stronger calcite deposition was detected at the unpaired angle where the paired rays contact (Additional file 1).

**Figure 2:**
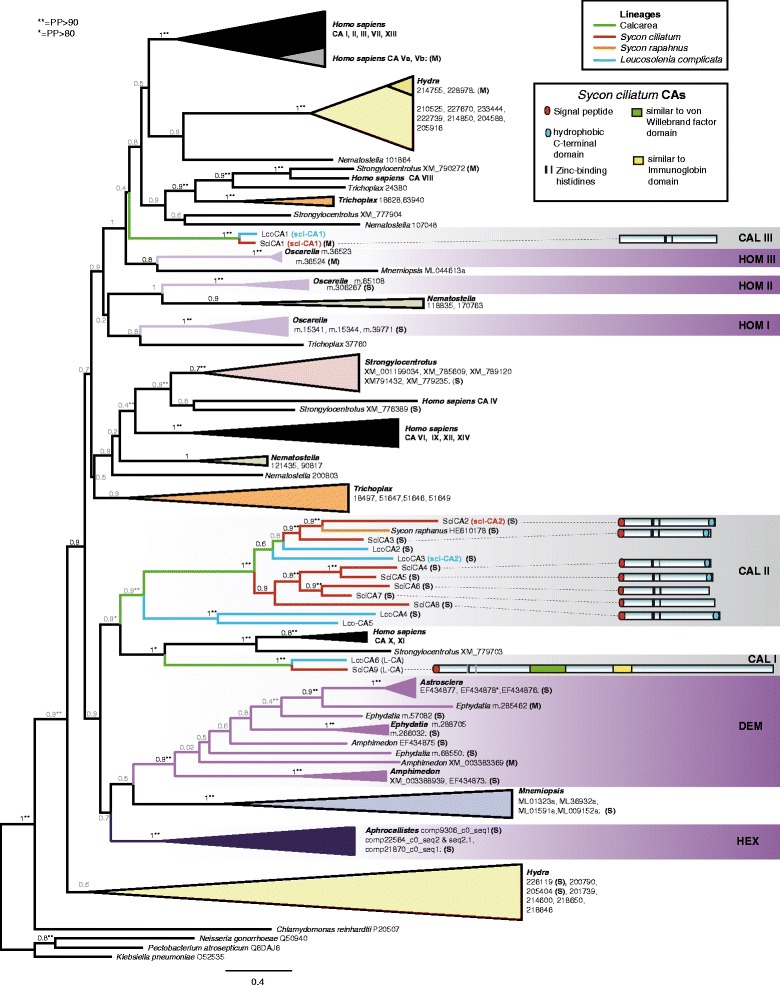
**Phylogenetic relationships (ML) of CAs and schematic representation of** ***S. ciliatum*** **CAs.** aLRT -support values are given at the branches and are shown in grey when BS support at the same nodes (values not shown) is below 50. PP support of the Bayesain phylogeny (Additional file 6) is indicated by *. (M): predicted mitochondrial localisation; (S): predicted signal peptide; CAL: Calacrea; DEM: Demsospongiae; HEX: Hexactinellida; HOM: Homoscleromorpha.

We calculated the spicule formation rates for three spicule types (the two diactines and triactines) by measuring the fluorescent signal for 15–22 spicules per type from small syconoid sponges incubated 18 h in calcein. Tetractines were omitted because their length was difficult to measure in spicule preparations. In triactines, paired and unpaired rays were considered separately. Growth rates differed considerably between spicule types (Figure 1d). The slender diactines showed the fastest growth (mean growth rate of 5.3 μm/h), followed by curved diactines (mean growth rate of 2.5 μm/h). The slowest growth was observed for triactines (mean growth rate of 1.4 μm/h for paired, 1.2 μm/h for the unpaired ray). Observations of complete sponges revealed that active spicule formation occurred all over the sponge body (Figure 1e), but was densely concentrated in two apical regions: (1) at the lower oscular region, where new radial tubes are formed (Figure 1e, f), and (2) in the proximal end of the palisade-like oscular slender diactines (Figure 1f,g).

### CA repertoire of calcareous sponges

Genome-wide screening revealed the presence of nine CAs in *S. ciliatum* (SciCA1-9, Table 1) and six CAs in *L. complicata* (LcoCA1-6, Table 1). They fall into three not closely related clades in our phylogenetic trees (CAL I-III, Figure 2), which are described in more detail below. Note that the numbering of CAs in both species does not imply gene orthology. The CA sequences were screened for the presence of signal peptides with SignalP 4.0 [31] and presence of the three zinc-binding histidines, considered to be required for the catalytic function of CA (e.g. [7,9]). Furthermore, we checked for terminal transmembrane domains using TMHMM-2.0 [32] and predicted the subcellular localisation of the protein using Target P 1.1 [33] (see Additional file 2 for protein sequences).

**Table 1:** **Properties of CAs in** ***S. ciliatum*** **and** ***L. complicata***

**CA**	**Accession**	**Coding sequence ID (Compagen)**	**Amino acid length**	**Signal peptide**	**Terminal transmembrane domain**	**Presence of 3 zinc binding domains**
*S. ciliatum*						
SciCA1 **(scl-CA1)**	LN609531	sctid70372	332	no	no	all
SciCA2 **(scl-CA2)**	LN609532	sctid21624	332	yes	yes	all
SciCA3	LN609533	sctid79452	316	yes	yes	all
SciCA4	LN609534	sctid52059	313	yes	yes	H1, H2
SciCA5	LN609535	sctid78781	322	yes	yes	all
SciCA6	LN609536	sctid91373	310	yes	no	all
SciCA7	LN609537	sctid82357	327	yes	no	all
SciCA8	LN609538	sctid21623	348	yes	yes	all
SciCA9 **(L-CA)**	LN609539	sctid19114	1171	yes	no	H2
*L. complicata*						
LcoCA1 **(scl-CA1)**	LN609540	lctid94802, lctid95538*	265	no	no	all
LcoCA2	LN609541	lctid78751	319	yes	yes	all
LcoCA3 **(scl-CA2)**	LN609542	lctid114957	326	yes	yes	all
LcoCA4	LN609543	lctid61203, lctid80506*	343	yes	no	all
LcoCA5	LN609544	lctid89007, lctid89853*, lctid88526**	281	no	no	all
LcoCA6 **(L-CA)**	LN609545	lctid17923	1172	yes	no	H2

*Potential allelic variant (>99% nucleotide similarity).

**Potential splice variant (>99% nucleotide similarity).

The calcarean clade CAL I contains one CA from each species (SciCA9 and LcoCA6, respectively), both of which are extraordinary large CAs (L-CAs), having 1171–1172 amino acids (AAs) compared to the remaining calcarean CAs proteins, which range between 265 and 348 AAs (Table 1). The L-CA possesses a signal peptide, suggesting the enzyme may be secreted. In the N-terminal CA domain of L-CAs, the first zinc-binding histidine is replaced by arginine and the third by glutamine. The same substitutions occur in the closely related (Figure 2) sea urchin CARP (Spu XM_779703) and in human CARPs CA X and CA XI. In the human CARPs these alterations of the zinc-binding sites are the cause of the catalytic inactivity [12]. Therefore, the L-CA proteins also probably lack CA activity. L-CAs also share additional substitutions with the urchin and human CARPs CA X and CA XI (four to five substitutions) and human CARP CA VIII (three substitutions) in 15 sites (Additional file 3) that were previously reported to be conserved among active CAs [9]. The C-terminal half of the L-CAs contain a domain with similarity to von-Willebrandt factor domains (Pfam [34]: Family VWA_2, Clan CL0128), and a domain with similarity to immunoglobin (Pfam [34]: Family Ig_2, Clan CL0011) (Figure 2, green and yellow boxes in scheme, respectively).

The calcarean clade CAL II contains seven *S. ciliatum* (SciCA2-8) and four *L. complicata* CAs (LcoCA2-5). With one exception from *L. complicata* (LcoCA5), all of these CAs have a signal peptide and, therefore, are potentially secreted*.* In *S. ciliatum*, five of these CAs additionally have a terminal hydrophobic transmembrane domain (SciCA2-5, SciCA8), which is also present in two CAs in *L. complicata* (LcoCA2, LcoCA3)*,* suggesting that these CAs are possibly bound to the extracellular membranes. The remaining CAs (SciCA6, SciCA7, LcoCA4, LcoCA5), which only show the signal peptide, might be free secreted forms. SciCA4 probably lacks CA activity because two of the three zinc-binding histidines are substituted.

The calcarean clade CAL III only contains one CA of each species, SciCA1 and LcoCA1, both lacking signal peptides and terminal transmembrane helices, so the CAs are cytosolic. A mitochondrial location is predicted by TargetP [33] only for the SciCA1, although with low certainty (Table 1).

### *In situ* hybridization and RNA-Seq analyses identify two sclerocyte-specific CAs

*In situ* hybridization (ISH)*,* and RNA-Seq (in *S. ciliatum*) was used to localize the spatial and temporal expression of the identified CAs in the two species. Because calcareous sponges are viviparous and only fully developed larvae leave the sponge, we could study developmental stages of *S. ciliatum* present within the adult tissue (see e.g. [27]). ISH experiments on *S. ciliatum* included tissues with oocytes, early embryonic stages (pre- and post inversion) and almost fully developed, but not yet released, amphiblastula larvae, as well as non-reproducing larger and smaller *S. ciliatum* individuals. Some experiments were conducted with post-larval and juvenile stages. For *L. complicata*, only adult tissue was used in ISH experiments.

**Figure 3:**
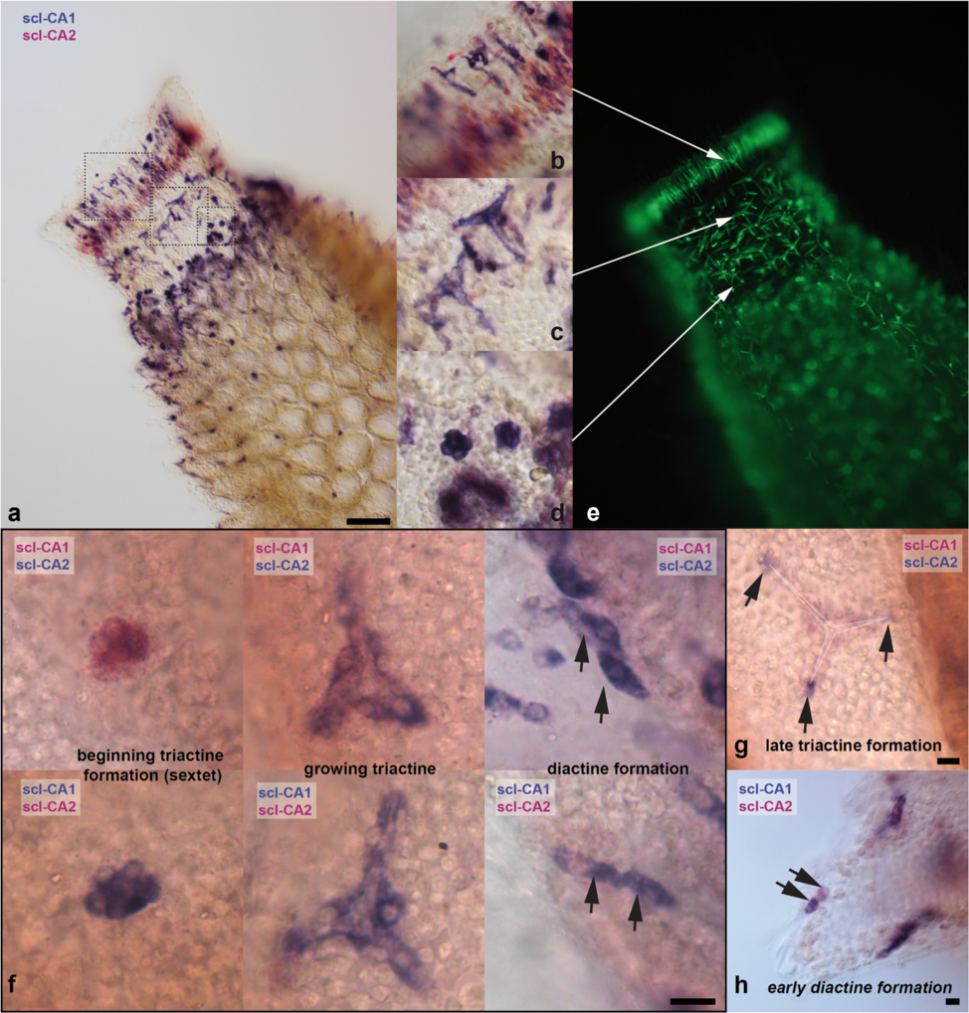
**Spicule formation patterns and expression patterns of scl-CA1 and scl-CA2 in** ***S. ciliatum.*** **(a-d, f-h)**: double ISH with differentially labeled probes. **(a-e)** small *S. ciliatum* individual, longitudinally cut and viewed from atrial side (scale bar: 100 μm). **(b, c, d)** Details from boxed areas in a. **(b)** Predominant scl-CA2 expression in elongated, diactine-forming sclerocytes. Note scl-CA1 expression in presumably tetractine-formation and in some elongated sclerocytes (compare to (e)). **(c)** scl-CA1 expression in tetractine forming sclerocytes. **(d)** scl-CA1 expression in sextets of sclerocyte cells, presumably early triactine formation of initial radial tubes. **(e)** Calcein staining showing spicule growth of 18 h. **(f-h)** Expression of scl-CA1 and scl-CA2 in sclerocytes of spicule formation in the radial tubes (scale bars: 100 μm). **(g)** scl-CA2 expression (purple) in founder cells. **(h)** Incidentally observed expression of scl-CA1 and scl-CA2 in two sclerocytes (presumably beginning diactine formation).

CAs with a role in spicule formation should be expressed in active sclerocytes (as already suggested by [22]), which should be most abundant in the regions of spicule formation detected in *S. ciliatum* in our calcein staining experiments (Figure 1e-g), and the buds of newly forming tubes in *L. complicata*. In both *S. ciliatum* and *L. complicata, s*uch patterns were observed in the expression of one intracellular CA (SciCA1, LcoCA1) and of one secreted/membrane-bound CA (SciCA2, LcoCA3). Additionally, the shape of expressing cells (Figure 3b-h, Additional file 4a,b) and their location in the sponge’s mesohyl (Additional file 4c) identifies the expressing cells as active sclerocytes, although the calcite spicules dissolved completely in the ISH procedure. We therefore refer to these genes as sclerocyte-specific CAs (scl-CAs): scl-CA1 (SciCA1, LcoCA1) and scl-CA2 (SciCA2, LcoCA3).

To further clarify whether the two scl-CAs are expressed in the same cells, double ISH with differentially labeled probes for each gene was performed to test the relation of scl-CA2 and scl-CA1 expressing cells in *S. ciliatum.* Scl-CA2 (SciCA2) was detected most prominently in elongated sclerocytes forming the slender diactines around the osculum (Figure 3a,b,e; compare Figure 1g). In the oscular region, scl-CA1 (SciCA1) was expressed in tetractine forming sclerocytes and sclerocyte sextets of newly forming triactines (Figure 1a), and other spicules forming in the region of the radial tube initiation (compare Figure 3e,f). In the earliest stage of triactine formation, only scl-CA1 is expressed in the six sclerocytes comprising the characteristic sextet (Figure 3f, left). From the sclerocytes of growing triactines in the radial tube, founder and thickener cells can be distinguished (compare Figure 1a), and all express scl-CA1 and scl-CA2 (Figure 3f, middle). In the double ISH, the less strong Fast Red labeling of scl-CA1 expression is not completely concealed by the purple scl-CA2 expression (Figure 3f, middle, top), suggesting that scl-CA1 expression in this stage is still high. Later triactine formation was not often observed, but incidental observation showed only scl-CA2 expression in the founder cells (Figure 3g).

Due to the density and position of diactines, it is much more difficult to identify sclerocytes of a single (dissolved) diactine, especially in later stages of diactine growth. However, closely associated sclerocytes in diactine-forming parts of the sponge show expression of both scl-CA1 and scl-CA2 (Figure 3f, right). In a few incidences, we also observed a differential expression of scl-CA2 and scl-CA1 in the later founder cell and later thickener cell, respectively, in a stage that seems to be a very early diactine formation (Figure 3h), but it remains uncertain if this represents a general transitional stage in all forming diactines.

**Figure 4:**
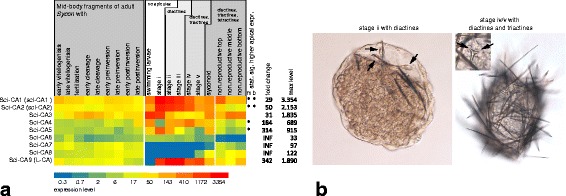
**Expression of CAs in different tissues and stages, juvenile stages. (a)** Heatmap representation of RNA-Seq expression profiles of CAs in different developmental stages and body parts of *S. ciliatum*. Expression level for each gene and stage or body part was calculated as a sum of the posterior probability of each read coming from that gene over all reads [36] and adjusted with size factors of the RNASeq libraries [37]. Statistically significant higher expression in the apical (top) region of the non-reproductive sponges in comparison to the middle or basal part is indicated by asterisks (‘m’ in comparison to the middle, ‘b’ in comparison to the base). The statistical significance was assessed using the adjusted p-value from DESeq package (p-value adjusted for multiple testing), padj ≤0.1.RNA-Seq. **(b)** Postlarval stages of *S. ciliatum* with only diactine (left) and diactine and triactine spicules (right). Arrows point at sclerocytes.

Illumina RNA sequencing (RNA-Seq) data confirms our ISH observations. It shows that in *S. ciliatum* the scl-CAs are, overall, continuously expressed in high levels and are significantly higher expressed in the top of the sponge compared to the middle or bottom part (Figure 2, see also [28]). Expression levels of the two genes are also highly correlated (R^2^ = 0.93). Furthermore, expression in spicule free stages was detected by RNA-Seq (larvae, settled postarval stage i, Figure 4a). ISH with larvae revealed weak expression in a ring of posterior-most micromeres (Additional file 4d, e). Micromeres make up the internal cell population of just settled larvae (postlarvae stage i), which still lack spicules but already highly express scl-CAs (Figure 4a). The first spicules to form are diactines, the only spicule type in postlarval stages ii-iii (Figure 4b). Here, the expression of scl-CAs is highest of all observed stages. In later stages, first triactines appear (iv-v, Figure 4b); then, after opening of the osculum and change of the juvenile morphology from round to vase-shaped, slender diactines form around the osculum (stage v). Tetractines were not observed (but were not specifically searched for) in juveniles, but were previously reported to appear first around the newly formed osculum [35], and in the later stages are present around the atrial cavity.

### Localization and expression of other CAs

According to RNA-Seq analysis, several CAs had low expression levels or no expression in studied stages, and we did not observe any expression pattern by RNA ISH in the studied stages of the sponge (SciCA5, SciCA6, SciCA8, Figure 4a). Because the active spicule formation detected by the calcein staining experiments requires the activity of CAs [22], we assume that the low-level expressed CAs are unlikely to be involved in the process. Expression of the presumably acatalytic L-CA SciCA9 (see above) peaks in post-settlement stages i-v, but was low in adult sponges (Figure 4a), where no ISH pattern could be observed. Because of the putative lack of CA activity (see above) and the fact that in spicule-forming adults expression is low, an involvement of this protein in biomineralization cannot be assumed.

Other CAs exhibited expression patterns with no relation to spicule forming sclerocytes (Additional file 4f-h). Besides the scl-CAs, SciCA3 also showed the highest expression levels in adult sponges. Here, the expression peaks in sponges with oocytes and early embryos (Figure 4), in which the transcripts of SciCA3 could be localized with ISH (Additional file 4f). We have not detected any cell-specific SciCA3 expression in the adult tissues and larvae, although RNA-Seq analysis indicates the presence of transcripts. Instead, choanocytes and, to a lower extent, pinacocytes, as well as all larval cells, developed uniform staining after a prolonged color reaction (Additional file 4f), consistent with the low-level ubiquitous expression of this gene, although qualitatively this staining is undistinguishable from background. A similar weak ubiquitous signal was observed for LcoCA2, while no expression patterns were detected for all the other LcoCAs except the scl-CAs in adult *L. complicata* sponges. SciCA7 expression was seen in exopinacocytes at the proximal base of the radial tubes of some adult sponges (Additional file 4g), but expression levels according to RNA-Seq were generally low in adult sponges (Figure 4a). ISH showed localized expression of SciCA4 in the macromeres of the larvae (Additional file 4h). Finally, SciCA9 (L-CA) was highly expressed only in juvenile stages i-v according to RNA-Seq (Figure 4i). The results of the iSH and RNA-Seq experiments are summarized in Table 2.

**Table 2:** **CA expression patterns of CAs in** ***S. ciliatum***

**CA**	**ISH**	**RNA-Seq**
SciCA1 (scl-CA1)	Sclerocytes, weak signal in ring of posterior-most micromeres in larvae	All stages (including spicule-free larva and postlarval stage i), higher expression in top region of sponge
SciCA2 (scl-CA2)	Sclerocytes, weak signal in ring of posterior-most micromeres in larvae	All stages (including spicule-free larva and postlarval stage i), higher expression in top region of sponge
SciCA3	Elevated expression in oocytes and early embryos; ubiquitous expression in other cell types including all larval cells.	All stages, higher expression in sponges with oocytes and early embryos, larvae and postlarval stage i
SciCA4	Larval macromeres	In larvae and postlarval stages ii-v, low expression in adult sponges
SciCA5	Undetected	Higher in larvae, postlarval stages i-iii, young syconoid sponge, low in other stages and adult sponges
SciCA6	Undetected	Very low expression during embryonic and postembryonic development
SciCA7	Proximal exopinacocytes between radial tubes	Low expression in adult sponges
SciCA8	Undetected	Low expression in adult sponges
SciCA9 (LCA)	Undetected	Highly expressed in postlarval-juvenile stages i-v, low expression otherwise

### Phylogenetic analysis

We reconstructed the relationship of CAs from sequenced genomes of all non-bilaterian phyla and additional transcriptomes of all four sponge classes (Additional file 5) with Maximum Likelihood (ML) and Bayesian methods to gain insight into the enzyme’s evolutionary history.

We did not recover a strictly eumetazoan CA clade as a sister group to demosponge CAs, which had been the result of earlier studies with fewer taxa [3,11,14,25]. Although approximate likelihood ratio test (aLRT) support values are high, bootstrap support is low, especially in the deeper nodes (Figure 2). The Bayesian phylogeny (Additional file 6) reflects these uncertainties by the presence of polytomies. Furthermore, some relationships differ from the ML topology, but mostly these alterative branchings are not highly supported. For example, in ML, a clade of *Hydra* CAs constitutes the sister group to all remaining CAs, while in the Bayesian tree (Additional file 6) we observe a polytomy at the base of Metazoa, with some of the mentioned *Hydra* CAs nested elsewhere in the tree. Most of the shallower clades occur in both trees, but in different relationship to each other, including the sponge clades. Further discussion will focus on the ML tree (Figure 2) because the model (LG + G) proposed by ProtTest 3 [38] was not available in MrBayes [39], which might in part be the reason for the discrepancies. Long branch artifacts could also influence our phylogenies (see e.g. [40]). Despite these difficulties and incongruences, the following important conclusions can be drawn from our phylogenetic analyses.

Sponge CAs are much more diverse than anticipated from previously reported demosponge CA sequences and, in our phylogenies, are not recovered in a single clade. Instead, demosponge CAs are a sister group to some ctenophore CAs, which together form the sister group to hexactinellid CAs (but compare the Bayesian phylogeny, in which a weakly supported monophyletic clade of CAs from these sponge classes is recovered, Additional file 6). However, CAs of both sponge classes are monophyletic. In contrast, the CAs of calcareous sponges and homoscleromorph sponges are more diverse, and three CA clades with no close relation to each other can be found in both classes (CAI-III and HOM I-III, respectively). Each of the three calcarean CA clades contains sequences from *S. ciliatum* and *L. complicata.* In clades CAL I and CAL III, only one CA gene of each species is included, with additional evidence of evolutionary conservation: scl-CA1 (clade CAL III) display sclerocyte-specific expression, while CAs of clade CAL I are characterized by the additional C-terminal domain (see above), and form a sister clade to the human CARPs CA X and CA XI and a sea urchin CARP (XP_779703). In contrast, relationships in clade CAL II are more complicated: Scl-CA2 of *S. ciliatum* and *L. complicata* both occur in this clade, but are not the closest related CAs. Instead, *S. ciliatum* scl-CA2 and the *Sycon raphanus* CA form a clade, to which SciCA3 is the sister group. Still closer than the *L. complicata* scl-CA2 (LcoCA3) is yet another *L. complicata* (LcoCA2, but see the Additional file 6 for a slightly alternative topology in the Bayesian phylogeny). This pattern demonstrates the difficulties in inferring CA function from only phylogenetic reconstructions, and suggests gene duplications and losses since the last common ancestor of the two species. From the phylogenies, it is obvious that scl-CA1 and scl-CA2 are not closely related and, therefore, most likely have been recruited independently in the biomineralization process. Neither scl-CA1s nor scl-CA2s have phylogenetic affinities with the *Astrosclera* CAs, which have been shown to be involved in the formation of the basal carbonate skeleton of this demosponge species [3].

**Figure 5:**
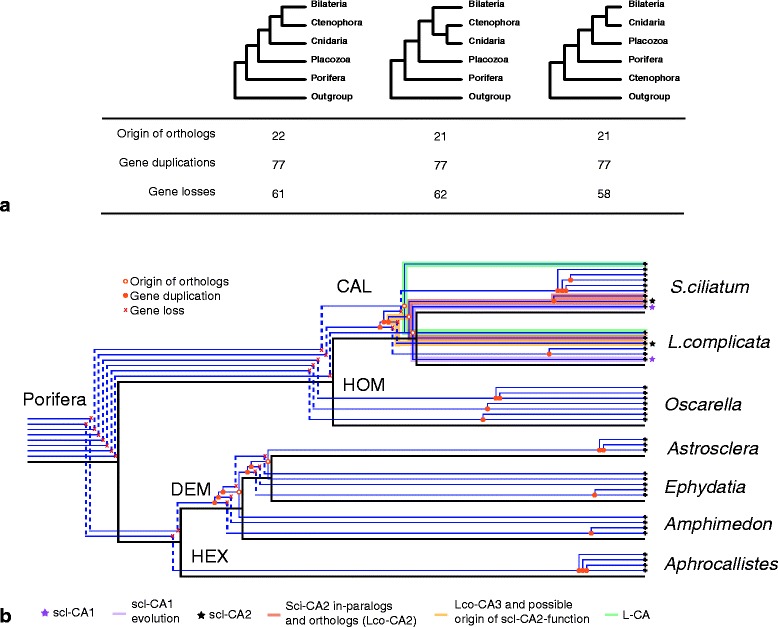
**CA evolution reconciled with three phylogentic hypotheses. (a)** Three different phylogentic hypotheses for the relationships of non-bilaterian phyla used for tree-reconcilation. The number of inferred ortholog origins (corresponds to ‘co-speciation’ in the JANE software), gene duplications and gene losses are presented for each evolutionary scenario. Note that the numbers are similar regardless the underliyng phylogeny of phyla. **(b)** CA-evolution in Porifera as obtained under all three phylogenetic hypotheses presented in **(a)**. Accordingly, eight CAs occurred in the last common ancestor of Porifera, of which several were lost in the different sponge lineages, followed by lineage-specific duplications. Three CA forms are in the last common ancestor gave rise to the three CA clades of Calcarea (CAL1-III, Figure 2). sclCA1 and L-CAs are recovered as orthologs in *S. cilatum* and *L. complicata.* A possible origin of ancestral scl-CA2 is indicated (orange). The monophyletic clades of demosponge and hexactinellid CAs (clade DEM and HEX, repectively in Figure 2) appears to be the result of substantial gene loss, followed by several duplications.

With the exceptions of Demospongiae and Hexactinellida, all included taxa possess more than one clade of CA, which must have arisen by CA duplications in early animal evolution. Also, species-specific or group-specific clades of CAs occur for all included taxa, suggesting a very frequent lineage-specific diversification of CA genes in animals. To gain further insight in CA evolution, we reconciled our CA phylogeny with two more recent hypotheses [41,42] and a more classical hypothesis about the relationships of non-bilaterian animals with methods provided in Jane 4 [43]. For each of the three phylogenetic hypotheses we visualized the potential CA gene histories, and found that in all cases frequent duplications and losses of CA genes can be observed (Figure 5a and Additional file 7). The reconstructions suggest that the last common ancestor of metazoans already possessed multiple CA genes, but the number of genes differs between the phylogenetic hypotheses (Additional file 7). Despite the underlying phyla-phylogeny, the presence of eight CAs in the common ancestor of sponges is reconstructed and identical gene histories within sponges are observed (Figure 5b). In each sponge class different ancestral CAs were lost, followed by a radiation from the remaining CA(s). At least three versions of CAs were present in the common ancestor of Calcarea, which were ancestral to L-CAs, scl-CA1 and to the CAs of clade CAL II, respectively. Clade CAL II includes scl-CA2, but within this gene lineage duplications and losses occurred after the common ancestor of *S. ciliatum* and *L. complicata* (Figure 5), which complicates ortholog assignment. According to our phylogenetic analyses and the tree reconciliation, scl-CA2 of *S. ciliatum* (SciCA2) and *L. complicata* (LcoCA3) are not of ortholog origin but instead might be out-paralog*s*, originating from a gene duplication that predated the speciation event (Figure 5b). The low support values and the differing topology of the Bayesian analyses, however, make unambiguous interpretations difficult. *S. ciliatum* scl-CA2 (SciCA2) and SciCA3 are in-paralogs, diverging from a lineage-specific duplication of an ancestral gene, and both are co-orthologs to LcoCA2 (Figure 5).

## Discussion

### Correlations and differences in scl-CA1 and scl-CA2 expression

Jones and Ledger [22] considered the possibility that the biomineralizing calcarean CA is cytoplasmic or secreted/membrane-bound. We present evidence that indeed two forms, one cytosolic and potentially mitochondrial form (scl-CA1) and one secreted/membrane-bound form (scl-CA2), are involved in spicule formation. The expression of both scl-CAs is highly correlated, and both scl-CAs are simultaneously expressed in triactine and diactine forming sclerocytes (Figure 3), suggesting a close interplay of the proteins in biomineralization. However, expression of the intercellular scl-CA1 precedes the initiation of secreted and membrane-bound scl-CA2, at least at the onset of triactine-formation in the sextet of sclerocytes (Figure 3f), but this stage might be very short-lived and obviously does not influence the strong correlation of the expression of the scl-CAs in the RNA-Seq data. It remains to be tested if, at this point of spicule formation, carbonate deposition has not yet started and if the presence of the extracellular scl-CA2 is required for its initialization. In late stages of spicule formation, expression of sclCA2 was only observed in founder cells (Figure 3g), which at this stage are no longer in physical contact to the thickener cells (Figure 1a).

Additionally, we observed a predominant scl-CA2 expression in the slender diactine forming sclerocytes situated around the osculum (however, some of these cells also showed scl-CA1 expression). It is noteworthy that the slender diactines are the fastest growing spicules in the sponge, as we found from the calcein staining experiments (Figure 1d). Furthermore, the texture of slender diactines of *Sycon* was reported to be almost identical to that of synthetic calcite and it was proposed that they contain no protein, in contrast to the other spicule types of the sponge [44]. If no additional proteins need be secreted to form slender oscular diactines, this may allow for faster growth, which can be driven by higher scl-CA2 expression levels in these sclerocytes.

### The role of the carbon source in understanding scl-CA function

The catalytic function of CAs (the interconversion of CO_2_ to HCO_3_^−^) can be involved in carbonate formation in animal skeletons in different ways, and is also dependent on the carbon source –metabolic CO_2_ or dissolved inorganic carbon (DIC) from the seawater– of the formed carbonate [16,45]. In corals, the origin of the carbonate carbon source is still debated and probably differs between species [11]. Consequently, the role of CAs in coral biomineralization is also not completely resolved. Secreted/membrane-bound CAs located at the calcification site can contribute to biomineralization in two ways, as, for example, was considered for a membrane-bound CA in the azooxanthellate coral *Tubastrea aurea*. If in the first case the skeleton’s carbon is largely of metabolic origin, activity of this CA can provide HCO_3_^−^ for the formation of CO_3_^2−^: in the presence of calcium, the produced CO_3_^2−^ can precipitate as calcium carbonate [16]. Alternatively, the authors argued that if HCO_3_^−^ (DIC) from seawater was the main carbon source of carbonate in *Tubastrea*, the extracellular CA could eliminate the protons that are released by the conversion of HCO_3_^−^ to carbonate. In this case this CA would catalyse the reaction of a proton and HCO_3_^−^ ion to H_2_O and CO_2_ [16].

As in corals, the carbon source of the calcareous sponge spicules is unknown. When the HCO_3_^−^ -concentration of seawater is artificially lowered below a certain threshold, no spicules are formed; this could be due to the fact that the amount of metabolic CO_2_ alone is not sufficient to maintain spicule formation [35]. However, under the conditions applied in this study, the lowering of the bicarbonate concentration in artificial seawater simultaneously led to acidification, which also may be the reason for of the lack of spicule formation [35]. Additional information about the carbon source of spicules can be drawn from stable isotope analyses of calcareous sponge spicules from species collected at the Great Barrier Reef [46]. In those samples, the δ^13^C-values differed among species, and were consistent with the separation of the two calcareous sponge subclasses. This observation contradicts the assumption that seawater is the sole carbon source because in this case similar values for specimens from the same environment would be expected. In fact, differences in the contribution of metabolic carbon and DIC to skeleton formation of different species might be the cause of the observed pattern. Therefore, we assume that metabolic CO_2_ contributes considerably to the carbon source of calcitic spicules in Calcarea.

### Potential function of scl-CA1 and scl-CA2 in biomineralization

**Figure 6:**
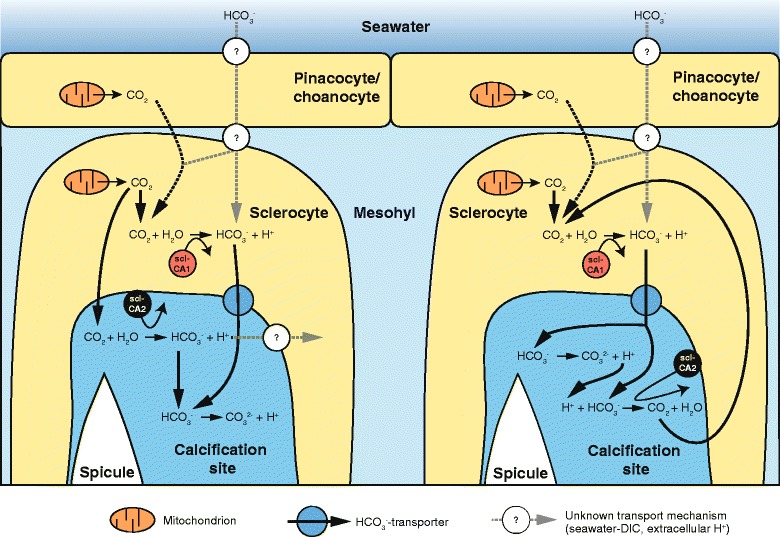
**Two scenarios for the potential function of scl-CA1 and scl-CA2 in spicule formation.** In both, scl-CA1 catalyses the formation of mainly metabolic CO_2_ to HCO_3_^−^ within the sclerocytes, and then transports it to the extracellular calcification site by a biacrbonate transporter. Here, scl-CA2 could also produce HCO_3_^−^ from CO_2_ diffused into the extracellular space (left). Alternatively, scl-CA2 could catalyse the reverse reaction, in order to remove protons that were formed by the reaction of HCO_3_^−^ to carbonate. The CO_2_ could then diffuse into the sclerocyte and again serve as substrate for sclCA1. In addtion to metabolic CO_2_, DIC (in form of HCO_3_^−^) might be taken up from the seawater, which could involve the activity of another CA, as had been suggested for corals [11]. The DIC transport form and mechanisms within the sponge tissue are yet unknown.

We have two scenarios for a potential interplay of scl-CA1 and scl-CA2 (Figure 6), which differ in the role of the secreted/membrane-bound scl-CA2. In both scenarios, the intracellular scl-CA1 transforms metabolic CO_2_ to HCO_3_^−^ within the sclerocyte, which then is secreted to the extracellular spicule formation site by a bicarbonate transporter [47]. The metabolic CO_2_ may not only be produced by mitochondria within the sclerocyte, but also by pinacocytes, choanocytes and other cells of the mesohyl, and diffuse into the sclerocyte. It is then possible that scl-CA2 at the calcification site transforms excess CO_2_ into additional HCO_3_^−^, which diffuses from the sclerocyte (Figure 6, left). In this case protons from the formation of carbonate could be actively removed, e.g. by a yet unknown Ca^2+^-ATPase, which in turn delivers Ca^2+^ to the calcification site, as has been proposed for corals [16,48]. Alternatively, the function of scl-CA2 could be to eliminate the protons by catalyzing the reaction of two HCO_3_^−^-ions and one proton to produce CO_2_ and H_2_O (Figure 6, right). The CO_2_ in turn could diffuse into the sclerocyte and serve as substrate for scl-CA1. This function of secreted/membrane-bound CAs was proposed for corals, if DIC in the form of HCO_3_^−^ was the main carbon source for the skeleton [11,16]. This is not a prerequisite in calcareous sponges as HCO_3_^−^ could also be provided by the activity of scl-CA1. However, DIC taken up by choanocytes or pinacocytes from seawater may contribute to the carbon pool, regardless of the function of scl-CA2, but neither the uptake mechanism nor the form of transportation is known. In corals, a membrane-bound CA from ectodermal cells is believed to be involved in DIC uptake by transforming HCO_3_^−^ into CO_2_, which diffuses into the cell [11]. According to the expression profiles, the only calcarean CA that showed continuous and high expression as would be expected for an enzyme with such a critical role is SciCA3, the closest CA to scl-CA2. The expression pattern of this gene appeared ubiquitous in adult tissues but elevated in oocytes and early embryos (see Additional file 4f), consistent with involvement of this CA in DIC uptake, where expression in choanocytes and pinacocytes would be expected.

### Evolution of CAs in sponges

To our knowledge, the phylogenetic analyses presented here is the first that includes CAs found in the completely sequenced genomes of all non-bilaterian phyla. Our phylogenetic trees strongly suggest that the CA repertoire of non-bilaterians, and especially sponges, is much more diverse than previously suggested by analyses that only included demosponge sequences. The last common ancestor of Metazoa is predicted to have already possessed a number of CAs (Additional file 7), instead of a single ancestral CA as had been suggested [3]. In contrast to this previous study, our results also suggest that the ancestor of sponges possessed a rich CA-repertoire (eight predicted ancestral CAs), of which several CAs were lost in the independent lineages that lead to the four recognized sponge classes. The extant CA-diversity in demosponges and hexactinellids is due to a reduction with a subsequent radiation of only a single ancestral CA lineage in both classes (Figure 5). The evolution of CAs in Metazoa was clearly driven by frequent gene diversification and gene loss events (Figure 5 and Additional file 7). This highly dynamic evolution makes CAs eligible for repeated independent recruitment in novel physiological roles, such as biomineralization, in different metazoan lineages, as we now also established for Calcarea. Even within calcareous sponges, duplications (and losses) occurred and, from all studied sponges the highest diversity of CAs was found in *S. ciliatum*. We could infer at least three hypothetical ancestral forms of CAs (one ancestral form for each calcareous sponge CA clade) in the last common ancestor of calcareous sponges from our phylogeny (Figure 2) and tree reconciliation (Figure 5): one intracellular form (ancestral to scl-CA1), one secreted or membrane-bound form (ancestral to scl-CA2 and related CAs), and one secreted form with C-terminal domains of unknown function (ancestral to L-CAs). The CA-domain of the latter is closely related to the two human CARPs, CA X and CAXI. The function of mammalian CARPs is unknown, but they are expressed predominantly in brain tissue and the binding of other proteins was suggested as a novel function of CARPs [10,12]. Interestingly, human CARPs CA X and CA XI, the potential urchin CARP and the CA-domain of L-CAs share specific amino acid substitutions in four to five positions that are conserved active sites of catalytic CAs [9]. The substitutions may be related to the altered functions of these proteins. CARPs related to human CAX and CAXI were previously known from other bilaterians [12], and the proposed close relationship of cnidarian CAs (Nematostella_107048 in Figure 2) to human CA X and CA XI [11] was not recovered in our tree. With our findings of L-CAs in calcareous sponges, it seems that these acatalytic CARPs (CA X, CA XI) have a much older origin at the base of metazoans.

The clade CAL II, which includes scl-CA2 (SciCA2 and LcoCA3) of both studied species, is the most diverse of the calcareous CA clades and gene orthology cannot be assessed easily due to duplications and losses (Figure 5). We found that SciCA2 and LcoCA3 are isofunctional paralogs involved in spicule formation. SciCA2 and SciCA3 are co-orthologs to LcoCA2, which originated from a lineage-specific duplication event after the separation of from *L. complicata* and *S. ciliatum*. According to our expression analyses, a functional differentiation of the two co-orthologs also occurred. The shared ancestral origin of the scl-CA2 genes is a gene duplication that predated the separation of the two species (Figure 5). Given the variety of temporal and spatial expression observed in *S. ciliatum* CAs of clade CAL II (SciCA2-8) the question remains as to what the function of the ancestral CA could have been. In our view, the independent recruitment of SciCA2 and LcoCA3 and CAs for biomineralization is very unlikely because of the similarities in the processes reported from both species [18,19] and the fact that their last common ancestor already must have processed calcite spicules (compare calcarean phylogeny in [49]). Conclusively, the involvement in biomineralization must be considered a plesiomorphic function of the CAs of this clade. The data, therefore, provides evidence that the last common ancestor of the calcareous sponges possessed two sclerocyte-specific CAs (Figure 5), which together are fundamental parts of the biomineralization toolkit in calcareous sponges. No pair of these intra- and extracellular scl-CAs are closely related to CAs involved in formation of the carbonate basal skeletons of the demosponge *Astrosclera* [3], supporting the view that CA-mediated carbonate formation evolved independently in calcite-spicule forming Calcarea and demosponges with a basal carbonate skeleton. The interaction of the two calcarean scl-CAs has to be considered as an evolutionary novelty that triggered the radiation of the extant calcareous sponges.

The increased taxon sampling, compared to previous studies of metazoan CA relationships (e.g. [3,11,14,25]), revealed that the deeper nodes are especially difficult to resolve. Our analyses lack strong BS and PP support in many nodes. Support as measured by aLRT values was in many cases higher and seemed to provide overoptimistic estimations in our tree reconstructions, especially for nodes with only low BS or PP support. aLRT have been shown to provide overoptimistic support values for data sets with a weak phylogenetic signal [50]. Therefore, phylogenetic analyses that only rely on SH-like aLRT values as measure of support (e.g. [25]) should be interpreted with caution if a weak phylogenetic signal is expected, as in the analyses of CAs. One has to consider that the resolution of nodes that, according to our results, date back to the origins and radiation of Metazoa. Reconstructing a supported phylogeny of such old evolutionary events is most unlikely when genomic and phylogenomic approaches utilizing hundreds of genes fail to provide a clear phylogenetic hypotheses about the relationships of the basal animal phyla (e.g., [40]).

### Relations of scl-CAs to *Sycon raphanus* CA

One of our identified scl-CAs in *S. ciliatum*, scl-CA2, is most similar to the only CA so far reported from calcareous sponges, *Sycon raphanus* CA [23,24], and likewise shares a signal peptide and a potential terminal transmembrane helix of the clade CAL II (Figure 2). A role in both spicule dissolution under Ca^2+^-depletion and coordinated spicule growth in in-vitro experiments has been suggested for the *S. raphanus* CA [23,24]. However, the authors showed an almost pervasive presence of the *S. raphanus* CA protein in many different cell types, including choanocytes (Figure 2C in [23]), similar to SciCA3 but not any other CA we have tested. Interestingly, *S. raphanus* CA is also detected in macromeres of an amphiblastula larva, coincidentally shown in one of the presented sections (Figure 2C in [24]), and is thus reminiscent of macromere-specific expression for SciCA4 (Additional file 4h), but not of scl-CA2 (Additional file 4e). Without knowing how many CAs are present in *S. raphanus*, it is difficult to say whether one *S. raphanus* CA is expressed in a combination of patterns from SciCA3 and SciCA4 or if the antibody is detecting several CAs simultaneously. In either case, in contrast to *S. ciliatum* and *L. complicata* scl-CA2 genes, the described *S. raphanus* CA gene expression does not appear to be sclerocyte-specific. Bearing in mind the surprisingly high diversity of calcareous sponge CAs demonstrated in the current study, especially within clade CAL II, and the fact that the genus *Sycon* is polyphyletic with *S. ciliatum* and *S. raphanus* not closely related [51,52], further studies should confirm the proposed function and localization of the *S. raphanus* CA for spicule formation.

## Conclusion

We identified one intracellular (scl-CA1) and one extracellular (scl-CA2) sclerocyte-specific CA as key components in biomineralization process of calcareous sponges. These enzymes are part of a complex repertoire of CAs in this sponge class. They differ fundamentally from the hitherto known sponge CAs from the class Demospongiae, for example by including acatalytic forms related to human CARPs CA X and CA XI. We demonstrate that the evolution of this enzyme family is very complex, both in terms of protein sequence and regulation of expression. Gene duplications apparently involved functional diversification with consequent differentiation in expression patterns. We propose that involvement in biomineralization was the original function of an ancestral enzyme of clade CAL II CAs that by gene duplication and functional diversification gave rise to the majority of the secreted/membrane-bound calcarean CAs with differing functions. Detailed expression studies, rather than sequence comparison alone, have proven most valuable in inferring the involvement of a specific CA in spicule formation, and the two identified genes can now serve as markers of active sclerocytes. Like corals and other calcifying marine invertebrates, calcareous sponges are potentially highly impacted by ocean acidification due to raising atmospheric CO_2_ levels [53]. Understanding the molecular processes in calcareous sponge biomineralization can help to estimate if or how Calcarea might respond to the changing environment.

## Methods

### Sequence identification and analysis

Genomic and transcriptomic sequences and RNA-Seq data were obtained as described previously [27-29]. CAs were identified using BLAST [54]. Sequences with >99% similarity were considered as allelic variants or splicing variants (Table 1).

The server versions of SignalP 4.0 [31], TargetP 1.1 [33] (both available at: [55]) were used to detect potential signal peptides and predicted subcellular location of CAs. Transmembrane domains were predicted with TMHMM-2.0 [32].

Amino acid sequences of CAs from additional taxa were obtained from GenBank [56] or identified by BLAST searches against data from sequenced invertebrate genomes [42,57-63] and transcriptomes [64] from data of publicly available sources: Compagen [65,66] and Metazome v3.0 [67] (Additional file 5). In *Hydra*, we excluded some proteins, which had additional domains and only partial CA-domains. Sequences were aligned with MAFFT version 7 [68] and sites for phylogeny were chosen manually by selecting regions of likely homology between conserved sites identified with Gblocks [69] (Additional file 8). ProtTest 3 [38] proposed the use of the LG + G model for maximum likelihood analysis (ML) under the AIC criterion. ML phylogenetic analysis was performed with PHYML [50], including 200 bootstrap replicates and SH-like aLRT to obtain support values. Bayesian inference was performed with MrBayes [39], using the mixed amino-acid model (because LG is not available), with a gamma parameter to account for rate heterogeneity. Two MCMCMC runs, with 4 chains each, were run for 10 million generations; every 1000^th^ tree was sampled. We omitted the first 40% of the sampled trees for the calculation of the consensus tree shown in Additional file 6. Tree reconciliation was performed in Jane 4 [43], using a simplified version of our ML phylogeny as “parasite” and different hypotheses [41,42] about phylum relationships as “host” tree. Sponge class and family relationships correspond to these and previous results [70]. In Jane 4 we used a population size of 2.000 for 200 generations with the ‘host switch’ parameter turned off.

### Sampling and calcein disodium staining

Specimens of *S. ciliatum* and *L. complicata* were collected and fixed for ISH as described previously [27]. For calcein staining, living specimens were transferred to a petri dish containing 30 ml of calcein disodium solution (12.5 or 125 mg/ml, Fluka) in seawater, and incubated at 14°C for 3–24 h. After cleaning by rinsing the treated sponges two times with fresh seawater, sponges were observed under fluorescence microscope (Nikon AZ100, using EGFP filter 41017) or fixed in 70% ethanol for later use. Carbonate deposited on the spicules during the incubation showed fluorescence due to the incorporated calcein. Spicules of sponges treated 18 h in calcein were isolated using bleach solution (containing 4% sodium hypochlorite), washed five times with deionized water and mounted on a microscopic slide. Two sponges were embedded in resin and sectioned with a Leica 1600 saw microtome as described previously [49]. Spicule growth was measured on spicule preparations (Figure 1b) for curved diactines and triactines. Growth of the more fragile slender diactines was measured in longitudinal sections (Figure 1c) because the spicules were easier to detect and remained undamaged. Spicule growth was measured in sponges incubated for 18 h at 14°C, which was preferred over a shorter 3 h incubation because the fluorescent spicules were very sparse in spicule preparations. To exclude spicules that began their formation long after the incubation had started, small completely fluorescent spicules were ignored. Incubations of 24 h were avoided so as to exclude measurement of spicules that stopped growing during the incubation. However, the possibility that some spicule elongation ceased before the end of the 18 h incubation cannot be excluded. Due to these considerations, the values presented in Figure 1d may underestimate the actual spicule growth rate.

### RNA *in situ* hybridization (ISH)

DIG-labeled specific antisense RNA probes were generated from 700–830 bp of the coding regions of all identified CAs of *S. ciliatum* CAs and of *L. complicata* from pooled cDNA from different developmental stages. PCR primers sequences are provided in Additional file 9. PCR products were cloned into the PCR4-vector (Invitrogen) and sequenced to determine the insert orientation. An additional PCR with the corresponding reverse vector primer and a probe-specific forward primer provided the template for the synthesis of DIG-labeled RNA probes (Dig-labeling kit, Roche). The probes were used in ISH of fixed tissue as described previously [27-29]. Fixed tissues included freshly fixed small *S. ciliatum* specimens and previously fixed larger sponges, some containing different developmental stages (oocytes, cleavage stages, pre- and post-inversion embryos and pre- release larvae). For ISH with *L. complicata*, only adult tissue was used and was treated in the same manner. During the ISH protocol, the carbonate spicules dissolved completely in most specimens. Double ISH was performed to compare expression of two selected genes in the same tissue by combining digoxigenin (DIG) and fluorescein labeled antisense probes of target genes. Gene expression was visualized by application of antibodies (FAB-anti-DIG and FAB-anti-fluorescein, Roche) and colorimetric detection (DIG: NBT/BCIP, fluorescein: Fast Red or INT/BCIP, both Roche). For documentation, ISH tissues were observed and stored in 75% glycerol. Selected ISH samples (complete small sponges or parts of tissue) were embedded in an epoxy-based resin and sectioned (5 μm thickness) using a Leica Ultracut microtome. The slide-mounted sections were documented using a Nikon DS-U3 microscope. Focused images of image stacks were generated with the Helicon Focus software (Helicon Soft).

## Ethics statement

No ethical approval was required for any of the experimental research described here.

## Availability of supporting data

The data sets supporting the results of this article are available in the Compagen repository [65], (genome assembly: SCIL_WGA_130802; coding sequences: SCIL_T-CDS_130802, LCOM_T-CDS_130802; proteins: SCIL_P-CDS_130802, LCOM_P-CDS_130802, http://compagen.org/datasets.html), in the European Nucleotide Archive [71], (CA- sequences: LN609531- LN609545, http://www.ebi.ac.uk/ena/data/view/LN609531-LN609545), and in the Open Data LMU repository [72], (phylogenetic dataset and phylogenetic trees: doi:10.5282/ubm/data.63, http://data.ub.uni-muenchen.de/63).

The genomic and transcriptomic datasets of calcareous sponges used here are described by Fortunato et al. [29].

## Additional files

Additional file 1:
**Calcein-stained triactine (18 h).** Arrow: Enhanced calcite deposition at the unpaired angle, which could frequently be observed. pa: paired actines; upa: unpaired actines. Additional file 2:
**Protein sequences of calcarean CAs.** Signal peptides are shown in lower case. The three zinc-binding histidines or substitutions in homologous positions are shown bold and underlined. Predicted hydrophobic stretches (potential membrane embedded domains) are underlined. For LCAs, additional identified Pfam domains are bold and italics. Additional file 3:
**15 active sites, previously reported to be conserved among active CA [** 9 **].** Note the shared substitutions of human (Hsa) CARPs (CAVIII, X, XI) and L-CAs. Two of the zinc-binding histidines are substituted by Argenine (R) and Glutamine (Q), respectively. +: active site hydrogen network, Z: zinc-binding histidine. (PDF 98 kb) Additional file 4:
**ISH of CAs in** ***L. complicata*** **(a, b) and** ***S. ciliatum*** **(c-h). (a,b) scl-CAs** ***L. complicata*** **: LcoCA1, LcoCA3).** Expressing cells occur more densely at buds (formation of new tubes, see arrows). (c) scl-CA2 expressing cells are located in the mesohyl (cho: choanoderm, mes: mesohyl, pin: pinacoderm). (d,e) Weak expression (left, grey arrows) of scl-CA1 and scl-CA2 in posterior-most micromeres, and expression in juvenile sponges (right). (f) SciCA3 expressed in oocytes and early embryonic stages which are present in the radial tubes of the sponge (left, middle). Longer development of the color reaction reveals ubiquitous (rather than cell-type specific) signal, which is difficult to distinguish from background staining in other tissues and larvae (right). (g) SciCA7 expression in basal exo-pinacocytes (ex-pin, arrow) on the base of radial tubes (section; atr: atrial cavity, rt: radial tubes). (h) SciCA4 is expressed in macromeres (black arrow) in larvae. All scale bars: 50 μm. Additional file 5:
**Accessions and sequence IDs of additional CA sequences included in phylogeny.**
Additional file 6:
**Bayesian phylogeny of CAs.** PP values given at the nodes, coloring and naming of clades corresponds to Figure 2. Additional file 7:
**CA evolution reconciled with three hypotheses of animal relationships.** Left: Classical concept, middle: “Coelenterata” [41], right: basal Ctenophora [42]. Filled dots: duplication events giving rise to paralogs; unfilled dots: duplication with speciation (origin of orthologs); dotted lines: gene loss in sisterclade. The evolution of scl-CA and LCA is highlighted. CAL: Calcarea, DEM: Demospongiae HEX: Hexactinellida; HOM: Homoscleromorpha; Aqu: *Amphimedon queenslandica*; Ava: *Aphrocallistes vastus*; Awi: *Astrosclera willeyana*; Emu: *Ephydatia muelleri*; Hma: *Hydra magnipapillata*; Hsa: *Homo sapiens*; Lco: *L. complicata*; Nve: *Nematostella vectensis*; Mle: *Mnemiopsis leydi*; Oca: *Oscarella carmela*; Spu: *Strongylocentrotus purpuratus*; Tad: *Trichoplax adhaerens.*
Additional file 8:
**Alignment (selected positions) for phylogenetic analyses.**
Additional file 9:
**Primer sequences.**

## Abbreviations

aLRT: Approximate likelihood ratio test
BS: Bootstrap
CA: α-carbonic anhydrase
DIG: Digoxigenin
ISH: RNA in situ hybridization
PP: Posterior probability
scl-CA: Sclerocyte specific carbonic anhydrase
