# Supplementary figures and images for: Calcareous sponge genomes reveal complex evolution of α-carbonic anhydrases and two key biomineralization enzymes
Source: BMC Evol Biol. 2014 Nov 25;14:230. doi: 10.1186/s12862-014-0230-z (PMC4265532; doi:10.1186/s12862-014-0230-z)

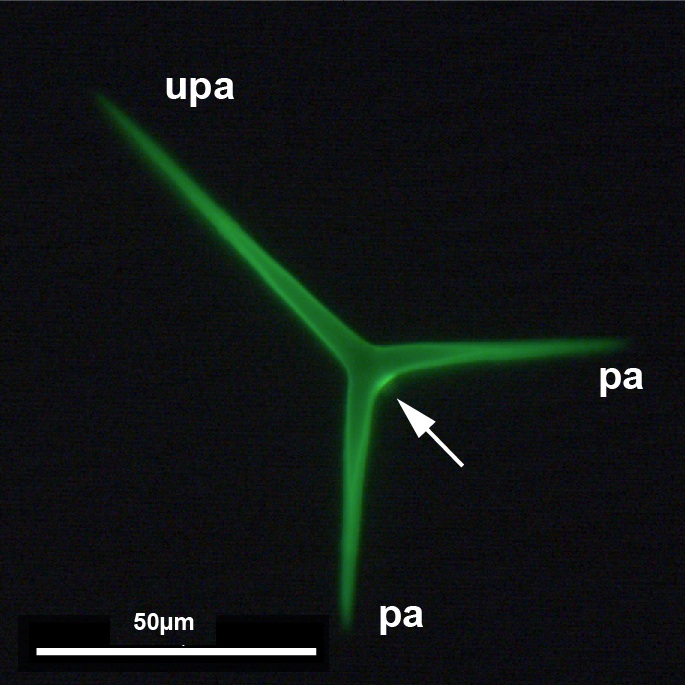

Supplement: Additional file 1: — Calcein-stained triactine (18 h). Arrow: Enhanced calcite deposition at the unpaired angle, which could frequently be observed. pa: paired actines; upa: unpaired actines. [file 12862_2014_230_MOESM1_ESM.jpeg]

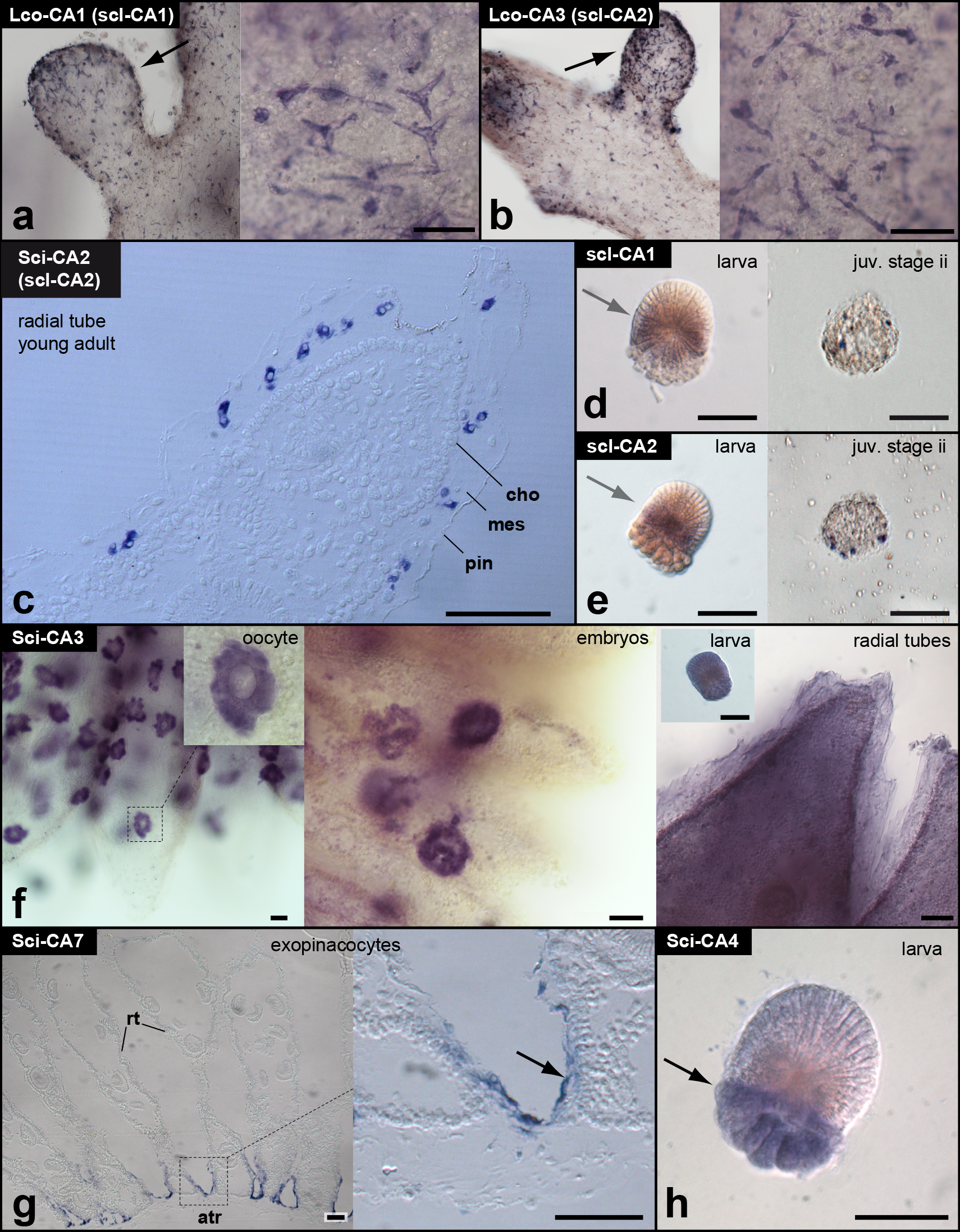

Supplement: Additional file 4: — ISH of CAs in L. complicata (a, b) and S. ciliatum (c-h). (a,b) scl-CAs L. complicata : LcoCA1, LcoCA3). Expressing cells occur more densely at buds (formation of new tubes, see arrows). (c) scl-CA2 expressing cells are located in the mesohyl (cho: choanoderm, mes: mesohyl, pin: pinacoderm). (d,e) Weak expression (left, grey arrows) of scl-CA1 and scl-CA2 in posterior-most micromeres, and expression in juvenile sponges (right). (f) SciCA3 expressed in oocytes and early embryonic stages which are present in the radial tubes of the sponge (left, middle). Longer development of the color reaction reveals ubiquitous (rather than cell-type specific) signal, which is difficult to distinguish from background staining in other tissues and larvae (right). (g) SciCA7 expression in basal exo-pinacocytes (ex-pin, arrow) on the base of radial tubes (section; atr: atrial cavity, rt: radial tubes). (h) SciCA4 is expressed in macromeres (black arrow) in larvae. All scale bars: 50 μm. [file 12862_2014_230_MOESM4_ESM.tiff]

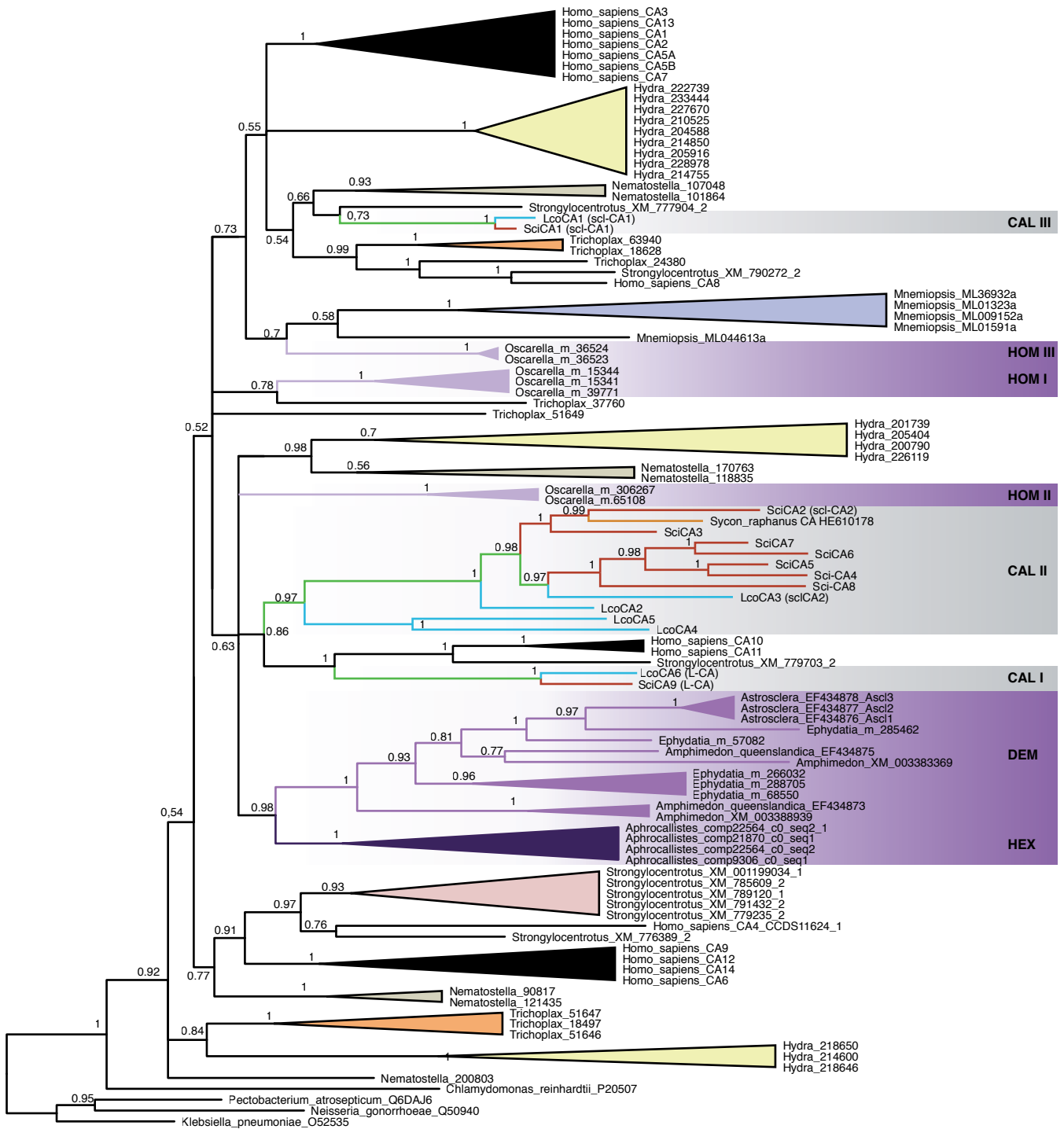

0.2

Supplement: Additional file 6: — Bayesian phylogeny of CAs. PP values given at the nodes, coloring and naming of clades corresponds to Figure 2. [file 12862_2014_230_MOESM6_ESM.pdf]

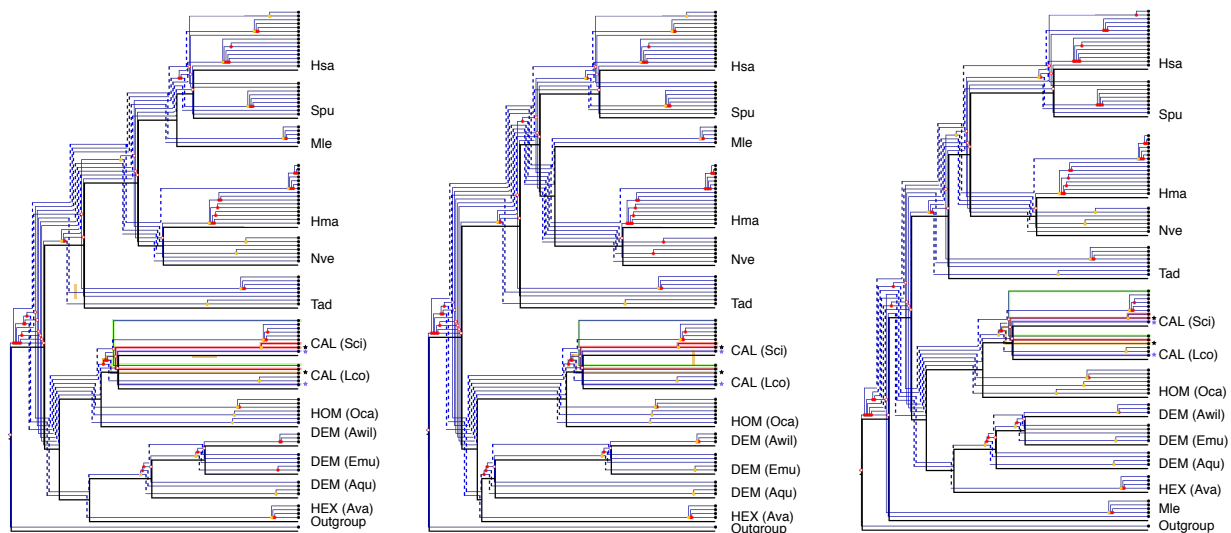

Supplement: Additional file 7: — CA evolution reconciled with three hypotheses of animal relationships. Left: Classical concept, middle: “Coelenterata” [41], right: basal Ctenophora [42]. Filled dots: duplication events giving rise to paralogs; unfilled dots: duplication with speciation (origin of orthologs); dotted lines: gene loss in sisterclade. The evolution of scl-CA and LCA is highlighted. CAL: Calcarea, DEM: Demospongiae HEX: Hexactinellida; HOM: Homoscleromorpha; Aqu: Amphimedon queenslandica; Ava: Aphrocallistes vastus; Awi: Astrosclera willeyana; Emu: Ephydatia muelleri; Hma: Hydra magnipapillata; Hsa: Homo sapiens; Lco: L. complicata; Nve: Nematostella vectensis; Mle: Mnemiopsis leydi; Oca: Oscarella carmela; Spu: Strongylocentrotus purpuratus; Tad: Trichoplax adhaerens. [file 12862_2014_230_MOESM7_ESM.pdf]
