# Supplementary material for: Calcareous sponge genomes reveal complex evolution of α-carbonic anhydrases and two key biomineralization enzymes
Source: BMC Evol Biol. 2014 Nov 25;14:230. doi: 10.1186/s12862-014-0230-z (PMC4265532; doi:10.1186/s12862-014-0230-z)
Supplement: Additional file 2: — Protein sequences of calcarean CAs. Signal peptides are shown in lower case. The three zinc-binding histidines or substitutions in homologous positions are shown bold and underlined. Predicted hydrophobic stretches (potential membrane embedded domains) are underlined. For LCAs, additional identified Pfam domains are bold and italics. [file 12862_2014_230_MOESM2_ESM.doc]

>SciCA1(scl-CA1)

MFLLGCVGEVVSRVGGAWRAPLLIPCSRVAKSTSSGAQRPTVAFLRSASQANSRFFSTSTSKKSSDMSWSHRPSLGCADVTEWQKHFPAAAGKRQSPIDLTKGEYNEELAGKALQISYKEARECTLVNNGHSVQVNFKPSIGGLTGGPVTDNFEIAQY**H**F**H**WGAGDDKGSEHTVDGKYYPNEL**H**LVHWNTSKFSSVSEAISEPNGLTVLGIFLEAGEENVELKKITDRFDKVKHCSQEIPIEDPICLKGLLPEDTKSFWSYDGSLTTPPCYESVNWIVFRKPITVSKEQLAAFRSLDEAKEGSDPEQHQALVDNYRPVCELNDRKLSTTFEE

>SciCA2(scl-CA2)

mitrlpavtavlamfvvcsmaHDPWNTDYTSQRGPLFWGQRPEFKMCGLGREQSPINIRRSSTIYQDFPPLAFELKSPIVHSNIENKGSATAAFPLSDIPILSGGPLGNRKYRVYNV**H**L**H**FGNYSFRAAEHAFDGVRTTGEF**H**IVTYDSRYPHIKAALGSGRRGALAVLGVMFEARNVSNIDMGVTNLIELSSNVTYKGDHYMTGIDFSNLVSEVDMGYYYAYNGSLTTPTCNEVVQWMVIDRIHYVLPETADLLLELKTGYRREHSIPIFGNTRPLQPLYGRKVLRSFGPVVTDVHDQGEEIVYSSADLVGPLGRVMLLALAAIATFVIKA

>SciCA3

mktafffaiavgmvaaasaaAKWSSSSRSPLGPQNWGSISGYELCGTGKSQSPININKASTTYKNHPALSFANGAGNIAFQMMNTGHAINALPSNPDGAPVLSGGPLGSATYRLYNI**H**I**H**FGNSTLKSSEHAFENTRTNGEF**H**IVTYKTTYGNISAAVGSGEAGALAVFGVLFDARNVSNTDSGITTFIDYASNLTYNGDSSTVSLPFPMMATTEDLKTFYTYSGSLTTPGCAEVVTWLVNDRIHYVTPDVMDQLAGLRSGNMSVPSYLLLDNTRPLQMLNGRVVERNFEAPSAASIVSVSLVSALVTIAASLKNL

>SciCA4

mkfiifmslavscsqvasQSWNYDPSSNIGPANWGNIPGFETCGTGTEQSPINIPTASAMYASYPPLNITSNATTDVYHLENVGDAPTGFAYGSSNTWFTGGPVGVTHYKLHSF**H**L**H**YGNSTYDGSEHAIDGMRTSAEL**D**VVFYNGSYQNLNQALASGDRDAIAIIAVLFSPSDDISHAHPGVTPLLNFFSQLTYRGDQSAEQLNFQDILRPEDITTFYTYYGSNSHPACNEQVVWMVMSQIQYYPPTMINALSGLLSSNRSEPRVEQIGNTRPLQPLNSRMIFQNFHTESSGSGLMTVSLVALLLSFLAASF

>SciCA5

mevkiiivtslaiacvqaASPGSNWNYDESSNIGPANWGNIQGFEGCGTGTEQSPINIQASSATYRSYPPLNVTSNSSTGTFRLENDGIGPIGIPDASNINYLTGGPLGDRQYRLQNF**H**L**H**YGNSTYEASEHAFDNMRTSSEI**H**FVFYNNIYANISAALDSKDRDALAVIGILFSTSMNVSHAHPSIVSILNFFAPLTYPGDTTTEQLDFSTVLRPEDVSTFYTYNGSLTTPSCNEQVVWLVMSQIQYYPPTVIDSLSSLLTTNRTQPPVGIIGNTRPLQPLNSRTIYQNFQNPTMASTTSGSGLMNISLFVMLLSFIVASV

>SciCA6

mevvlllslcitavyaSGGSPQELWNYNPDSPLGPAGWGNISGFKTCGNGTDQSPINIPKASAAYMTYPALNVTSSPSNASFYFYANNDAAVRADHFDTTSVSITGGPLDSEQYSIDNF**H**I**H**FGNSTSMSSEHSVDGSRTAGEL**H**VVFFNSKYSSLRKALASGDSDAEAVISILFNVASNASSAHPGLETILRHFSTLTYSAFGDIHSIDFATLLRPSDLTSFYTYKGSQATPGCNEQVIWMVMNQIQYVLPASLNSLSSGLNPVSGLPSAFIFGGNARPLQSLNDRTIYQNFPMTSGPPTVATSTAAPQ

>SciCA7

mellvllslaitttfaFDPSQQTWSYISSSNIGPASWGTLSGFETCGTGTEQSPINIPTASTDYQSYPPLNVTDSSSSAYFHFENNGAAAEADPEGTTVVYLTGGPLGSERYRIQNF**H**I**H**FGNSTFAASEHAVDGSRTAGEL**H**VVFFKATYPNIADALASGDRDALAVIGMLFDVGSDASLAHPGLTTMLNHFSNLTYPGFSVVQSLDFATLLRPSDLTSFYTYEGSLTTPGCNEQVVWLVLSQVQYVLPASLDALSGLLCTSPTQTPAIPVFGNARPLQSLDVRRIFRNFQATTSGPNTTSSAPGQLKVSLFALFISVLAVCTHWM

>SciCA8

mqlaiatllstllvytsnaGSGGWDHDPTSMIGPKYWGNVSAICRDGRKQSPINIVPPSTIYQPYPALNLTVPEGNAVFKFGNGGHDVSGHPVVSSGGNILLRGGPLGAAYKFHNI**H**I**H**YGNKSMPASEHSVERARTTGEL**H**AVFYNSSFTTLSDAAADNSSSAIAVIGIRFAAGLWVNESYYHAGLQSFFENVASLTYKGNETTHSFNMSDLLRPEDIDRFYTYEGSLTTPNCQETVQWLVMEKTQYVRSTLIDAISHFRSGNTLTSKLIIGNARPVQALNGRTIYRNFQIPQSEPSIAPSTWRTGNASASTTPVVSTATSGVAPIAAAPVVLTTTVLVFTFATLKN

>SciCA9_(L-CA)

mtrtgesvhlllfslvivtgisaEHAQLPCTEWSYADQDNWPDTHCTGNSQSGINIETISTAFHPDFSSLAIDTAESGVSVMLHNDGHHVVIRGNGLEVLGFLSGGPLYSDQTLFSIVEA**R**F**H**FGVVVDGAVVRGSDHLVDGNSYAAEL**Q**FIAFNTMYPSMKVALTQPGAVAIVSVLFELGDASNNVDLNMFLDTVASEVTYEGNSFGLDGINLRQLLPLSISDYYTYSGSFSWPKCSEDVTWLVMSQTLPITLDQMNRLQGVFSTRRRQNPAIPMAGNTRQTQELNSRQIISSFPTSQAVSLGCNHQNEQISLGQTHEFFSPDASDPRSTRGGN***DIIFLVDQSGSSRIRRLHSWLRQADFAGKLDTALTGLGFSNNQFGMVGFGQDQGGPATTIEVGGAGQLLGSAEDLQTAFQGLTSRGSREDGYAAIQLALRAYDLRPNAHCILLMLTDEGRDNLAEDLNYDLMVSELNSKKCVLHVA***VHERFASGMRPHCGRRPFHRHAFGMDAEKHSIIKHSVEEFQVVPWAGYARLRSGNKNTHTAYVQLAWATGGSAFNVIEITDVGSADRVQAAATFSNILAQLRMRDLQRYSCQRCRCTFAGQVDCRRCDEDFPPQA***VVVAEPATVFLGDSGTLRCIASGHPAPTIAWQRPDGSTNLPPGVTPSGSGDRLNIRSVTERNCFTCVADGGRLGEN***EAMGCVEVQGVIPVPELTQPQSTNTGGCATFTCTSSGYPEPRITLTVNSSDCVVSGNTVSCDNLQATACVTCNSENRLGSGEDTKCVNVFASCPGNIHHNSWRRQFSPDASIPADNMHLVIVFDESRTMLTERGALPDIITGLDTALMAAGFGVSIENKYSLVGFAGSAAPGGKYIPVGNGICGGAAEVGALASTLGRDGRTEDGYSGINVALQDATQCDTGPNSQLMILIMTDEDRDVLRGVGESFTYSTIVQSLRTANARFSAIVKQQYRNFVTGQDAYGLDSKGQTFIIAGGGQEFELTPPGTGGPIRDSGFGTTEQDYVELAQDTGGCVFDVLSVRNAGRRPGFLAALSACLSEQLSAERVRPLCQECLCNSGELQCNNLPRITDRAQCTSPSDVNLGVSVRSDPPFAVDNQQMTLFCESSNERAVAEWVPHPIDAFQIGRDLHLSACAAKSAPSYMCVLRTGDGQDSDSISVGL

>LcoCA1 (scl-CA1)

MAWSHRPSTGCADVSEWQKHFPHAAGKQQSPIDLTKGEYSKDLASKPLKLEYKDARECSMVNNGHSVQVNFKPAVGGVTGGPVTDNFELAQY**H**F**H**WGAGDDKGSEHTVDGKFYPNEL**H**LVHWNTSKFSSVGDAISEPNGLTVLGIFLEVGEENVELKKITDHFDKVKHAGQEFDLVDPICIKGLLPSDTKSLWSYDGSLTTPPCYESVNWIVFKKPLSVSKEQLAAFRSLSETKQDADPEKQENLVDNYRPVCPLNDRKLRTTFK

>LcoCA2

mkvsfailllcevaavlvsaQWSYNEANGDGPSMWGSNPAYYTCGNGTSQTPIDIVQSTTTYMSYPTLQFMNNSDPVRFRLMNDGHGIAALRMTSSNNGNPILMGGPLGTDTYRIYNY**H**I**H**FGNSTFVAAEHAFDGYRTTGEL**H**VVTYNTKFANIGLAVGSGERNALAVVGILFNATASTPSPTNPILDSMISDAANLTYKDDGIDIAIDFRQLVPNTDLRNYYTYSGSLTTPGCAEVVTWLVMSGTVQITNQQIQSLSSLVSGTQASNNTYPLYGNTRPLQMLNGRPVMRSFMLSSACHLSASFSILAAISAFLVLFL

>LcoCA3 (scl-CA2)

masiigaicvlsamlavasgAGKSPPWNYDDRTQYGPSNWGNHPTTAACKDGTTQSPININKLSTSYRRGLPPLRFDFTNNNVAWKLRNDGHALSALPVNPYKAPTLSGGPLGNSIYRVYNY**H**L**H**FGSEYMPASEHSFNDRRTTGEL**H**VVTYNTRYASIGDAIASKDFNALAVVGTLFHISDSQISHPGLLSTLFYSNNLTSPNDAAVVTVPMNNLVTNEIMEKYYMYPGSLTTPGCAETVQWLVLDTVAYVPTASIRDVTDLRSKQIGSLPIAPIFGNTRPLQALNSRVVTRNFQLGFSQESSAAQFSASLALLVAAALTVLMAL

>LcoCA4_translation

mkglcvmaivaliglqtatgYTRQDLSCGGRMDYSKPVCHNIPKHAHKKCDVYNQWSHHLFGTSQKCWGKLNPACRGMRQSPININEHKVEPNHNYGDLCITGPHHLKVHIHNTGHDLQAKLDESSSRATLVTGGPLGNKKYRVLQF**H**F**H**FASHPGGKGSEHSINCHFSDIEM**H**IVLQNVAYGSFDVAKDHRDGLSVIAVMLSEDVRPNTVSNAMRNNPSWSQYYINTLIYYASLRKHCDLHEVPGNTRFSLFHLLPSDYARNYYAYGGSLTTPPCSESVSWIIMRTRFHINRYHLNLLKDVSLLSRYHRGFEPMSQNKRNLQLLNNRKVYYPAGHGRCPSRG

>LcoCA5_translation

GSNQPCWGQLDCSCNGLRQSPINIPRRVEVDASLPDLCMDQSSTWLSGTIGNNGHSITAKLSPGGVQGSSAFGGPLKGTFKIEQF**H**F**H**FASSGYGSEHAIRGQKAAAEM**H**IVMFNKRYRNVAEALKHCQGLAVVGVLLSSDRRHADARNAWGEDPHNTQRFWIQLIQELSQHRTCRHDNRWTSINKRLKQLLPANYHRDYYTYEGSLTTPPCTESVQWIVMKKPFYVPARYFRALGAIQDSHHHRMSGNRRNIQDLNGRKVRQPTSSNRSPSCPRRRSWEN

>LcoCA6_translation L-CA

mgltgasylllfsaafsgfcgiygKNQDSCEDWSYRDQENWPAQCYKSPIDINRAQASFHPNFSPLAIDTASVNTIIANNGKHIVIHGGDDHQVLGFLSGGPLFSETALYGIVEA**R**F**H**FGLRIDGRSMGGSDHTISGKSYDAEL**Q**FIAYNTMYPNVEVALLQPNGIAIVSVLFEEGERTDNLDLNMFLDMAASEVKFHGNDFSLDGINLRQLLPDNIGEYFTYNGSFTWPRCSDPVNWIVMSNTMTITAEQLSNLQGVYSTRRGQSPEEPMAMNTREIQDHPDNIGRAVVASFPAGRAVVLDCTHEREQIPLGHTRQFSTPDTSDPRSSRAGN***DIIFIVDQSGSSRVRRLHRWLKDTEFPMKLDAAFNDNGFENNRFGLVGFGHDLSGQASTIPVGQGGQLIGSAADLQRAMQGLISRGNREDGYAAIQLALRAYDFRPDAHTILMLMTDEG*RD*ALAPDLDRAAMETILVDGKTTLH***AVLHEHFRSGMRSGCRPRHRGHDHALGMGPMKHAFLRHAGADFEVAPWLGYPRLRSAHTNTHDAYTELAWSSGGGVFDILSVVNSGTQDRNNVANSLSNMFAQMRLWDFQRFNCQRCTCKFSAEVECSRCDEDFPPQA***VVEAAPTVFLGEAAQLRCVASGHPPPGVAWQQTDGSSQLPSGVTVEGDTLRIASVTQRNCFTCVASGGRQGER***ESTGCVEVQGVIPEPVVSQPSPINAGASAQFTCTSTGYPEPELAFETDNDNCVADGTTVTCSNLQRRTCVTCVSSNRLGSGSDTQCVDVYASCEGGVRHNSCRRRFTPDASIPADNIHLVVLFDESRTMLNERAALGRIVAGIDDTLRSAGFGVNIDNKFSLVGFAGSSAPQGRSISVAGGVCGSPTELEAATNALSEDGRTEDGYSAIDVALSTVTGCDTGPNSQLVLMLFTDEDRDTLRGFGESITFDSTLQSLQAANARFVAVVKQEYRDTPARRSAYGLDSLGKAFVVTDGGASFEVSASGSPVRDSGYGTTERDYVDLARQTGGAFFNTLVIRDEGRLAGFIAAVAFSLSEQLASERVRPLCQECCCSNGSPNCTNMPRITAQAACMSPPAVELSTSVSSTPSFAMHAEEMTLFCGGDNENAITQWEPHPVEAFQTGSQLHFASYTRAASTTFVCTQRIDNGMDSGSLTLDAALGALGK
