# Supplementary material for: Calcareous sponge genomes reveal complex evolution of α-carbonic anhydrases and two key biomineralization enzymes
Source: BMC Evol Biol. 2014 Nov 25;14:230. doi: 10.1186/s12862-014-0230-z (PMC4265532; doi:10.1186/s12862-014-0230-z)
Supplement: Additional file 3: — 15 active sites, previously reported to be conserved among active CA [ 9 ]. Note the shared substitutions of human (Hsa) CARPs (CAVIII, X, XI) and L-CAs. Two of the zinc-binding histidines are substituted by Argenine (R) and Glutamine (Q), respectively. +: active site hydrogen network, Z: zinc-binding histidine. (PDF 98 kb) [file 12862_2014_230_MOESM3_ESM.pdf]

```
                 + +ZZ+++Z ++ ++
Hsa CA II        SNQHHEHEHVYTPWR
SciCA1(scl-CA1)  ...............
SciCA2(scl-CA2)  ..N............
SciCA3           ..N............
SciCA4           ..S.....D..S...
SciCA5           ..N............
SciCA6           ..N........A...
SciCA7           ..N............
SciCA8           ..N............
LcoCA1(scl-CA1)  ...............
LcoCA2           T.N............
LcoCA3(scl-CA2)  ..N............
LcoCA4           ...............
LcoCA5           ...............
SciCA9(L-CA)     ..ER.D..QI.S...
LcoCA6(L-CA)     ..ER.D..QI.....
Hsa CA VIII      ..ER.....I.....
Hsa CA X         ..ER....QI.....
Hsa CA XI        ..ERL...QI.S...
Spu XM_779703    ..ER....QIS....
```
