# Supplementary material for: Calcareous sponge genomes reveal complex evolution of α-carbonic anhydrases and two key biomineralization enzymes
Source: BMC Evol Biol. 2014 Nov 25;14:230. doi: 10.1186/s12862-014-0230-z (PMC4265532; doi:10.1186/s12862-014-0230-z)
Supplement: Additional file 5: — Accessions and sequence IDs of additional CA sequences included in phylogeny. [file 12862_2014_230_MOESM5_ESM.pdf]

Group	Organism	Data source	Data version	Accession/ Sequence IDs
Chordata	*Homo sapiens* (CA I-CA XIV)	NCBI	n.a.	NP_001729, NP_000058, NP_005172, NP_000708, NP_001730, NP_009151, NP_001206, NP_005173, NP_004047, NP_001207, NP_064563, NP_001208, NP_001209, NP_940986, NP_036245
Cnidaria (Anthozoa)	*Nematostella vectensis*	JGI (http://genome.jgi.doe.gov/)	N.vectensis v1.0 models	90817, 101864, 107048, 118835, 121435, 170763, 200803
Cnidaria (Hydrozoa)	*Hydra magnipapillata*	Metazome (http://www.metazome.net)	proteome	200790, 201739, 204588, 205404, 205916, 210525, 214600, 214755, 214850, 218646, 218650, 222739, 226119, 227670, 228978, 233444
Ctenophora	*Mnemiosis leidyi*	NHGRI (http://research.nhgri.nih.gov/ mnemiopsis/)	Mnemiopsis leidyi transcriptome 2.2	ML009152a, ML01323a, ML01591a, ML044613a, ML36932a
Echinodermata	*Strongylocentrotus purpuratus*	NCBI	n.a.	XM_001199034, XM_776389, XM_777904, XM_779235, XM_779703, XM_785609, XM_789120, XM_790272, XM_791432
Placozoa	*Trichoplax adhaerens*	JGI (http://genome.jgi.doe.gov/)	Triad1_best_transcripts	18497, 18628, 24380, 37760, 51646, 51647, 51649, 63940
Porifera (Calcarea)	*Sycon raphanus*	NCBI	n.a.	HE610178
Porifera (Demospongiae)	*Amphimedon queenslandica*	NCBI	Aqu1	EF434873, EF434875, XM_003383369, XM_003388939
Porifera (Demospongiae)	*Astrosclera willeyana*	NCBI	n.a.	EF434876, EF434877, EF434878
Porifera (Demospongiae)	*Ephydatia muelleri*	Compagen (http://compagen.org)	EMUE_T-PEP_130911	m.57082, m.68550, m.266032, m.285462, m.288705
Porifera (Homoscleromorpha)	*Oscarella carmela*	Compagen (http://compagen.org)	OCAR_T-PEP_130911	m.15341, m.15344, m.306267, m.36523, m.36524, m.39771, m.65108
Porifera (Hexactinellida)	*Aphrocallistes vastus*	ERA archive of the University of Alberta (http://hdl.handle.net/10402/e ra.38025)	AV4-1_Trinity.fa	(comp22564_c0_seq2*, comp3738_c0_seq1, comp7621_c0_seq1), (comp9306_c0_seq1*, comp9306_c0_seq2, comp9683_c0_seq1), (comp22564_c0_seq1*, comp22564_c0_seq3), comp21870_c0_seq1
Proteobacteria	*Klebsiella pneumoniae*	NCBI	n.a	O52535
Proteobacteria	*Pectobacterium atrosepticum*	NCBI		Q6DAJ6
Proteobacteria	*Neisseria gonorrhoeae*	NCBI	n.a	Q50940
Chlorophyta	*Chlamydomonas reinhardtii*	NCBI	n.a	P20507

*identical sequences in sites selected for phylogeny given in parenthesis, only the marked sequence was included in analysis
