# Supplementary material for: Calcareous sponge genomes reveal complex evolution of α-carbonic anhydrases and two key biomineralization enzymes
Source: BMC Evol Biol. 2014 Nov 25;14:230. doi: 10.1186/s12862-014-0230-z (PMC4265532; doi:10.1186/s12862-014-0230-z)
Supplement: Additional file 9: — Primer sequences. [file 12862_2014_230_MOESM9_ESM.pdf]

Additional file 9: Primer sequences for RNA-ISH probe generation.

Primer name	Sequence (5'-3')
SciCA1fw	ACAGTTGCCTTTCTCCGCTCAG
SciCA1rv	CCAGCTGCTCCTTTGAGACAGT
SciCA2fw	AGTTCAAGATGTGCGGTCTCGG
SciCA2rv	TAGCATCACACGACCGAGAGGA
SciCA3fw	CATCAACATCAACAAGGCCAGCAC
SciCA3rv	GCGATGGTAACGAGAGCACTGA
SciCA4fw	CATGTAGCCAAGTGGCGAGTCA
SciCA4rv	GCGCATTAATCATTGTGGGCGG
SciCA5fw*	AACGGAGCAGTCGCCTATCAAC
SciCA5rv*	TAGTGCGGGAATTGAGTGGCTG
SciCA6fw	GACGTGCGGCAATGGAACAG
SciCA6rv	TGATTGCAGCGGACGAGCA
SciCA7fw	CAACAGCGAGCACAGACTACCA
SciCA7rv	CTGTCCTGGAGCTGAAGACGTG
SciCA8fw	ATTGGGCCGAAGTACTGGGGTA
SciCA8rv	TGATGAGCTTGGAGGTCAGGGT
SciCA9fw	CTCATCCCTGGCCATCGACACAG
SciCA9rv	TGCTTGACTGGTCGGGAATGAAGA
LcoCA1fw	GTGCAGATGTGAGTGAGTGGC
LcoCA1rv	AGGTTTTCTTGCTTCTCGGGGTC
LcoCA2fw	ACGTACATGTCCTACCCGACGC
LcoCA2rv	GGAGGCAGATAGATGGCAGGCA
LcoCA3fw	GGAGCAATTTGTGTGCTGTCCG
LcoCA3rv	CAGTTGGGACGTAAGCCACAGT
LcoCA4fw	CCATTGTGGCCCTTATCGGGTT
LcoCA4rv	TAGTAGTTGCGGGCGTAGTCCG
LcoCA5fw	CCATGTTGGGGACAGCTGGATT
LcoCA5rv	ACACGACGGACTTCGATTGGAC
LcoCA6fw	GCTTTCTCAGGCTTCTGCGGTA
LcoCA6rv	ATCTCTCGCGTGTTCATTGCCA

* amplifies also Sci_CA4
